# Bilateral double osteotomy in severe torsional malalignment syndrome: 16 years follow-up

**DOI:** 10.1007/s10195-013-0260-0

**Published:** 2013-08-29

**Authors:** Francesco Leonardi, Fabrizio Rivera, Alessandra Zorzan, Syed Mohsin Ali

**Affiliations:** 1 Orthopaedic Surgery Departement, SS Annunziata Savigliano Hospital, Azienda Sanitaria Locale CN1, Via Ospedali 14, Savigliano, Cuneo Italy; 2 Orthopaedic Surgery Departement, SS Savigliano Savigliano Hospital, Azienda Sanitaria Locale CN1, Via Ospedali 14, Savigliano, Cuneo Italy; 3 Barnet and Chase Farm NHS Trust Hospital, London, UK; 4 Via Servais 200 A 16, Turin, Italy

**Keywords:** Torsional malalignment syndrome, Femoral torsion, Tibial torsion, Osteotomy

## Abstract

**Background:**

Torsional malalignment syndrome (TMS) is a well defined condition consisting of a combination of femoral antetorsion and tibial lateral torsion. The axis of knee motion is medially rotated. This may lead to patellofemoral malalignment with an increased Q angle and chondromalacia, patellar subluxation and dislocation. Conservative management is recommended in all but the most rare and severest cases. In these cases deformity correction requires osteotomies at two levels per limb.

**Materials and methods:**

From 1987 to 2002 in our institution three patients underwent double femoral and tibial osteotomy for TMS bilateral correction (12 osteotomies). All patients were reviewed at mean follow-up of 16 years.

**Results:**

At final follow-up no patients reported persistence of knee or hip pain. At clinical examination both lower limbs showed a normal axis and a normal patella anterior position. Pre-operative femoral version measurement showed an average hip internal rotation of 81.5° (range 80°–85°) and average hip external rotation of 27.2° (10°–40°). Thigh–foot angle measurement showed an average value of 38.6° (32°–45°). At final follow-up femoral version measurement showed an average hip internal rotation of 49° (range 45°–55°) and average hip internal rotation of 44.3° (20°–48°) (Figs. 1, 2, 3, 4, 5, 6). Thigh–foot angles measurement showed an average value of 21.6° (18°–24°) outward.

**Conclusion:**

We recommend a clinical, radiographical and CT scan evaluation of all torsional deformity. In cases of significant deformity, internally rotating the tibia alone is not sufficient. Ipsilateral outward femoral and inward tibial osteotomies are our current recommendation for TMS, both performed at the same surgical setting.

## Introduction

Torsional malalignment syndrome (TMS), also called miserable malalignment syndrome [1] represents one of the more complex problems that orthopaedic surgeons treat. This condition usually consists of a combination of femoral antetorsion and tibial lateral torsion. The axis of knee motion is medially rotated. This may lead to patellofemoral malalignment with an increased Q angle and chondromalacia, patellar subluxation and dislocation. Since correction of the malalignment would require osteotomies at two levels per limb, operative correction is seldom justified. Conservative management is recommended in all but the severest cases. Clinical indications for operative correction are: a child older than age 8 years, medial hip rotation beyond 85° and lateral tibial torsion of 30° or more [2].

## Materials and methods

A retrospective review was undertaken identifying a group of patients who pre-operatively presented with patellofemoral pain or snapping hip during ambulation due to rotational deformity of the lower limb. From 1987 to 2002, 12 surgical corrections for lower limb rotational deformities in nine patients were performed. In four patients unilateral femoral osteotomy was performed, in two patients unilateral tibial osteotomy was performed. The remaining three patients were treated by a bilateral double (femoral and tibial) osteotomy. Inclusion criteria for bilateral double osteotomy was: maximum age of 30 years, minimum hip internal rotation of 80°, thigh–foot angle more than 30°, persistent anterior knee pain. Patients with a single bone deformity were excluded from the study. The group of three patients submitted to bilateral double osteotomy for TSM was studied by a retrospective chart review and a clinical and radiographical follow-up. All patients were female with an average age of 20.6 years (range 17–24). All had complaints of inwardly pointing patellae and significant anterior knee pain exacerbated by activity. Two patients reported trochanteric pain after flexion-extension movement of the hip. In these two cases clinical examination revealed an external snapping hip. One patient had undergone previous surgical tibial epiphysiodesis. All patients were pre-operatively clinically evaluated by femoral version and thigh–foot angle measuring. Standard radiographic anteroposterior, lateral, and Merchants views of the knee and anteroposterior views of both limbs with the patellae anterior were obtained. Pre-operative CT scans were performed in all patients to evaluate femoral anteversion, tibial external torsion and the relationship between these variations in all candidates for lower limb osteotomy. Pre-operative planning was performed by angle of version and torsion calculating according to the Lerat et al. [3] method.

In all cases surgical correction of TMS was obtained by double corrective osteotomies, externally rotating the distal part of the femur and internally rotating the distal part of the tibia. In all cases the femoral and tibial procedures were done at the same operative session. Femoral osteotomy was performed first. Intraoperative assessment of correction was made with the use of guide pins inserted proximal and distal to the osteotomy and perpendicular to the bone. The distal part of femur was then externally rotated according to a predetermined degree to achieve approximately equal medial and lateral femoral rotation. After this first step the patella appeared less internally deviated but the foot was more externally rotated. Tibial osteotomy was then performed. Internal derotation of the distal part of the tibia was performed by adding up degrees of performed externally rotated femoral osteotomy to degrees pre-operatively calculated, to achieve an internal rotating tibial correction. The goal should be to achieve a thigh–foot angle of <20° [2, 5]. For femoral deformity a proximal metaphysis osteotomy was performed in one patient and a distal metaphysis osteotomy was performed in two. For tibial deformity a proximal metaphysis tibial osteotomy was performed in all cases. In the case of proximal femoral osteotomy, a lateral approach to the trochanteric region was used to reduce the chance of medial circumflex artery lesion. The osteotomy was done at the lesser trochanter level. A 90° blade plate is applied after the appropriate derotation. In the case of distal femoral osteotomy, a lateral approach to the distal femoral metaphysis was performed. A 6- to 8-hole plate is applied after derotation. The tibial osteotomy was done 3 cm below the growth plate, at tibial tubercle level. Staples were used for stabilization in all cases. No additional soft-tissue procedures were performed directly to affect patellar tracking. Partial weight bearing was started after 4 weeks; full weight bearing usually was started after 6 weeks.

All three patients with six double osteotomies were available for a follow-up. Subjectively and clinically, all of the patients were reviewed at an average of 16.3 (range 20–12) years after surgery. The following parameters were checked: patient satisfaction, reported knee or hip symptoms, gait, clinical femoral anteversion and tibial torsion, return to sport activity. In all patients radiological (CT) femoral anteversion and tibial torsion were studied after surgery. The need for informed consent was waived by the ethical committee since rights and interests of the patients would not be violated and their privacy and anonymity would be assured by this study design. The study was performed in accordance with the ethical standards of the 1964 Declaration of Helsinki as revised in 2000.

## Results

Pre-operative femoral version measurement showed an average hip internal rotation of 81.5° (range 80°–85°) and average hip external rotation of 27.2° (10°–40°). Thigh–foot angle measurement showed an average value of 38.6° (32°–45°) outward. Pre-operative CT scans measurement showed an average femoral anteversion of 37.8° (29°–52°) and an average tibial outward rotation of 47° (42°–54°). All surgical procedures were performed without complication of infection, neurological problems, or compartment syndromes. All osteotomies healed without complications.

**Fig. 1:**
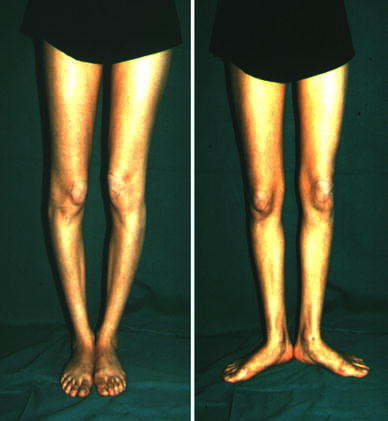
Patient DT. Clinical photographs of a 23-year-old girl. Inwardly pointing patellae are seen with feet parallel (*left*). Anterior pointing patellae are seen with outwardly rotated feet (*right*)

**Fig. 2:**
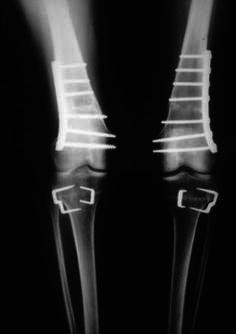
Patient DT. Radiograph after correction: supracondylar osteotomy of the femur fixed with plate and screws and proximal tibial osteotomy fixed with staples

**Fig. 3:**
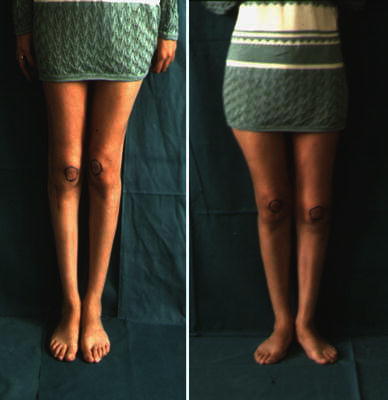
Patient DT. Clinical photographs after torsional defect corrections. Standing position with feet parallel (*left*). Standing position with outwardly rotating feet (*right*)

**Fig. 4:**
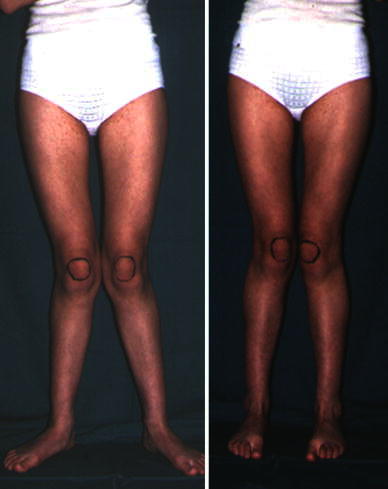
Patient GS. Clinical photographs of a 17-year-old girl. Anterior pointing patellae are seen with outwardly rotated feet (*left*). Inwardly pointing patellae are seen with feet parallel (*right*)

**Fig. 5:**
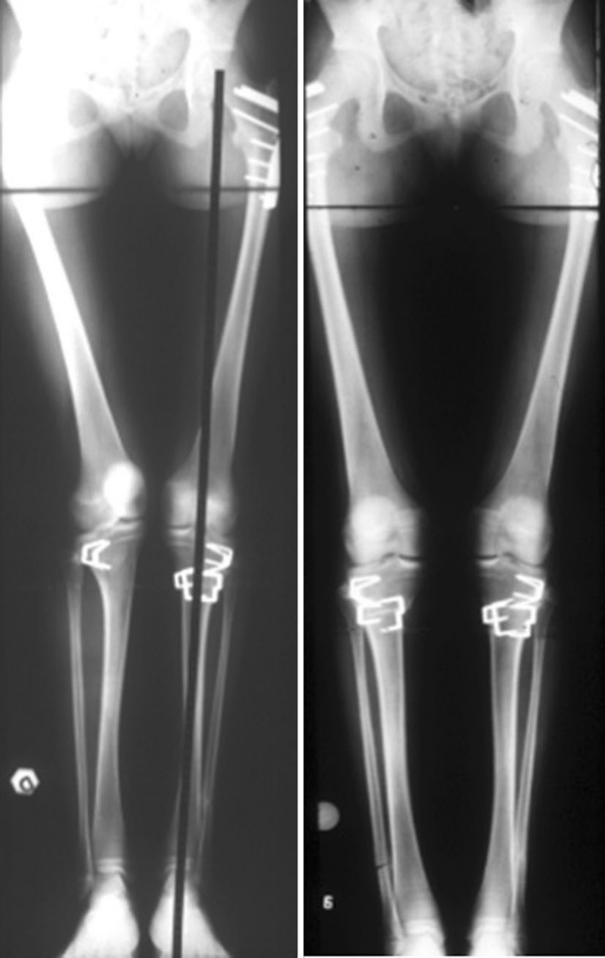
Patient GS. Radiograph after correction: proximal osteotomy of the femur fixed with blade-plate and screws and proximal tibial osteotomy fixed with staples

**Fig. 6:**
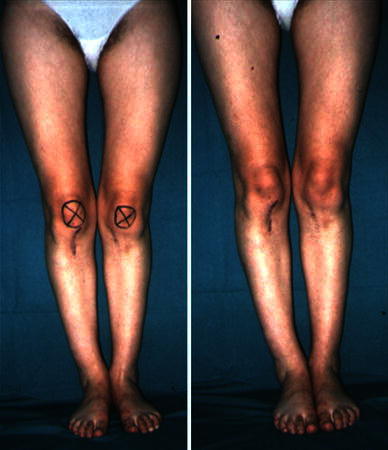
Patient GS. Clinical photographs after torsional defect corrections. Standing position with outwardly rotating feet (*left*). Standing position with feet parallel (*right*)

Post-operative CT scan measurement after corrections showed an average femoral anteversion of 21° (12°–30°) and an average tibial outward rotation of 26.3° (30°–32°). At final follow-up femoral version measurement showed an average hip internal rotation of 49° (range 45°–55°) and average hip internal rotation of 44.3° (20°–48°) (Figs. 1, 2, 3, 4, 5, 6). Thigh–foot angles measurement showed an average value of 21.6° (18°–24°) outward.

In all patients a good result was obtained (Table 1). At final follow-up all patients reported progressive marked relief of symptoms during normal activity after surgery and consequent progressive increase in function. Two patients began a non-agonistic sport activity 1 year after second limb surgery (volleyball, swimming). No patients reported persistence of knee or hip pain. At clinical examination both lower limbs showed a normal axis and a normal patella anterior position. Patellar tracking during knee flexion and extension was asymptomatic within the femoral groove. All knee joints were stable and had full range of motion. No snapping hip or trochanteric pain was present in all cases.

**Table 1:** Pre- and post-operative deformity values

Patient	Side	Age	Sex	P-op FIR	P-op T-FA	P-op CT FA	P-op CT TOT	Year of surg	Final FU	FU FIR	FU T-FA	FU CT FA	FU CT TOT
DT	L	23	F	82	35	34	50	1991	20	45	24	25	24
	R	24	F	80	38	34	48	1992	19	50	22	22	20
FR	L	20	F	80	36	28	46	1995	16	50	20	12	30
	R	22	F	82	32	29	45	1993	18	50	18	15	30
GS	L	17	F	85	45	52	42	1997	12	45	22	30	32
	R	18	F	80	40	50	54	1998	13	55	24	22	32
Mean value		20.6	–	81.5	38.6	37.8	47	–	16.3	49	21.6	21	28
Standard deviation		2.80	–	1.97	4.50	10.51	4.18	–	3.26	3.76	2.33	6.57	4.89

*P-op FIR* pre-operative clinical femoral intra rotation, *P-op T-FA* pre-operative clinical thigh–foot angle, *P-op CT FA* pre-operative femoral antiversion measured by CT scan, *P-op CT TOT* pre-operative tibial outward torsion measured by CT scan, *Year of surg* year of surgery, Final *FU* final follow up (years), *FU FIR* clinical femoral intra rotation at follow-up, *FU T-FA* clinical thigh–foot angle at follow-up, *FU CT FA* post corrections femoral anteversion measured by CT scan, *FU CT TOT* post corrections tibial outward torsion measured by CT scan

## Discussion

When there is increased internal torsion of the femur or when there is excessive external torsion of the tibia, inward pointing of the knee is most commonly increased. This excess inward point of the knee introduces an excess force pulling on the medial soft tissues and compressing the bone laterally. This may result in pain, patellar instability and chondromalacia and it can evolve to arthritis [1, 4, 5].

In 1957 Somerville [6], who successfully treated eight patients, aged 5–21 years, with double-level osteotomies, identified the torsional malalignment syndrome and proposed its surgical correction. Ever since this report, only a few experiences have been reported in the literature. Staheli [2] stated that since correction of malalignment would require osteotomies at two levels per limb, operative treatment is effective in relieving the knee pain in these patients. In 1993 Mylle et al. [7] reviewed 17 patients treated for lower limb torsional deformity using three different fixation techniques. The authors suggested external fixator use because it allowed early mobilization and partial weight bearing, but they have not reported the number of unilateral double femoral and tibial osteotomies.

Meister and James [8] reported on the operative treatment of six patients who had malalignment syndrome. All six had debilitating knee pain. The authors reported satisfactory results in all cases, performing single tibial rotational osteotomies. Intraoperative torsional correction of severe torsional tibial defects was obtained with an internal rotation average of 19.7°. Torsional femoral deformity was considered mild in all cases and it was not corrected.

In a group of nine patients with 13 involved extremities, treated for symptomatic severe torsional malalignment of the lower extremity and associated patellofemoral pathology, double osteotomy was performed only in seven cases [9]. In the two patients who had double-level osteotomies bilaterally, the femurs were derotated first, and the tibias 4 months later. In three patients who had double-level osteotomy unilaterally, the femoral and tibial procedures were done at the same operative session.

The most numerous group of patients was retrospectively reviewed in 2004 [10]. Fourteen patients with 27 limbs were treated by ipsilateral outward femoral osteotomy and inward tibial osteotomy. Thirteen limbs were also treated with a concomitant ‘mini-open’ lateral retinacular release. No persistent complications were seen. Subjectively and clinically, good results were shown at an average of 5.2 (range 2.0–12) years after surgery.

A possible complication due to tibial rotational osteotomy is peroneal nerve palsy [10]. Since the peroneal nerve runs around the fibula, increased tibial torsion of the proximal tibia during internal rotation osteotomy may result in overextension of the nerve or entrapment in the intercompartmental septum between the anterior and lateral muscle compartments. Bruce and Stevens [10] reported one case of bilateral peroneal nerve neuropraxia and irritation that required exploration and external neurolysis in a patient treated by bilateral double femoral and tibial osteotomy. This patient underwent bilateral femoral osteotomy and bilateral inward tibial rotational osteotomy during two separate surgical operative sessions. Peroneal nerve complication was observed after a later surgical setting. For this reason they suggested performing limb correction at the same surgical setting, since the effect on the peroneal nerve seems to be lower.

In our cases the site of femoral osteotomy varied. Femoral rotational osteotomy may be performed either proximally or distally. Proximal osteotomies are frequently intertrochanteric, and distal osteotomies are most commonly supracondylar. One study [11] reports that intertrochanteric osteotomy allows more accurate correction of the deformity and decreases the need for post operative immobilization.

Despite the conclusions of the aforementioned study we agree with demonstration of the same good results obtained by use of both proximally and distally osteotomies reported by Kay et al. [12].

Fixation may be provided by a variety of devices or crossed pins [12]. In our experience plate osteosynthesis for femoral fixation and staples osteosynthesis for tibial fixation provided good results without complications. Long-term endurance can be obtained now by using a low-profile locking compression plate with an angular-stable connection between screws and plate.

We recommend a clinical, radiographical and CT scan evaluation of all torsional deformities. We suggest treating deformities that are significant and are believed contributory to patellofemoral alignment. In our opinion, in cases of significant deformity, internally rotating the tibia alone is not sufficient, because these patients rarely have sufficient passive external rotation of the femur to accommodate the operatively internally rotated tibia. Ipsilateral outward femoral and inward tibial osteotomies are our current recommendation for TMS, both performed at the same surgical setting.
